# Routes of Travel

**Published:** 1916-10

**Authors:** 


### Routes of Travel.

With special reference to poliomyelitis, we wish to call attention to discrepant statements regarding distribution of disease by routes of travel. Most of the statements in the recent literature, speak of poliomyelitis as following routes of travel. Some of the older ones state the contrary. Practically any hamlet is on a route of travel. When it said that a disease follows a route of travel, the elements of time, progressive, not necessarily straight following of a thoroughfare of some kind, bulk of travel, should by implication be significant. The superstition of the Wandering Jew in regard to cholera, is a classic example. When we find a small, scattered village in Steuben Co., off all main routes of travel, having the same number of cases of poliomyelitis as Buffalo, and more than many cities of large size, when we note that Philadelphia and other cities to which rival roads from New York runs trains "every hour on the hour," have had comparatively few cases, when we consider the absence of cases in Vermont and the fact that only three have developed in New Hampshire and 14 in Maine, all states largely sought by New Yorkers for vacations; when we reflect that the first autochthonous Buffalo case occurred after an imported, Brooklyn case, but without proximity and too soon for this source of infection, we feel that Routes of Travel are distinctly not a factor in the spread of this disease.

